# Sialyl Glycan Expression on T Cell Subsets in Asthma: a correlation with disease severity and blood parameters

**DOI:** 10.1038/s41598-019-45040-2

**Published:** 2019-06-20

**Authors:** Yu-Liang Yeh, Wen-Chia Wu, Reiji Kannagi, Bor-Luen Chiang, Fu-Tong Liu, Yungling Leo Lee

**Affiliations:** 1 Institute of Epidemiology and Preventive Medicine, College of Public Health, National Taiwan University, Taipei, Taiwan; 2 Institute of Biomedical Sciences, Academia Sinica, Taipei, Taiwan; 3 School of Medicine, College of Medicine, Taipei Medical University, Taipei, Taiwan; 4 Department of Pediatrics, National Taiwan University Hospital, Taipei, Taiwan

**Keywords:** Polysaccharides, CD4-positive T cells, Regulatory T cells, Asthma

## Abstract

Memory T helper (Th) and regulatory T (Treg) cells play key roles in asthma. Certain sialyl carbohydrate determinants for selectins profoundly affect the migratory properties of memory Th cells, and the suppressive function of Treg cells. Previous studies have shown that the proportion of CCR4^+^ memory Th cells expressing sialyl 6-sulfo Lewis X (Le^X^) is elevated in asthma patients. We aim to investigate the roles of different sialyl glycans on T cell subsets in asthma. Using flow cytometry, we assessed the expression of three sialyl glycans, sialyl 6-sulfo Le^X^, cyclic sialyl 6-sulfo Le^X^, and sialyl Le^X^ on memory Th and Treg cells, in the peripheral blood of asthmatic children. We also assessed the relationships between glycan-expressing cell percentages and asthma clinical parameters. Compared with controls, asthmatic children showed higher proportions of memory Th cells expressing sialyl Le^X^ and sialyl 6-sulfo Le^X^. The proportions of memory Th cells with sialyl 6-sulfo Le^X^ and cyclic sialyl 6-sulfo Le^X^ expression in asthmatic children correlated with absolute eosinophil count and IgE level, respectively. Children with moderate-to-severe asthma had lower numbers of sialyl Le^X^ positive Treg cells. Our study suggests that sialyl glycans on T cells may play important roles in the pathogenesis of asthma.

## Introduction

Asthma is classically considered a T helper 2 (Th2)-associated inflammatory disease, and asthma patients have elevated serum Immunoglobulin E (IgE) levels and eosinophilic inflammation^1^. Emerging evidence, however, has also emphasized the roles of other T cell types, as well as their specific subsets, in asthma pathophysiology^2^. Homing receptors such as CCR4^3,4^ and CCR7^5^ were found to be critical in determining the migratory properties of different T cell subsets such as memory T helper (Th) cells and regulatory T (Treg) cells in asthma. More importantly, our previous preliminary study revealed an increased ratio of CCR4^+^ memory Th cells which express a sialyl sulfoglycan called sialyl 6-sulfo Le^X^, from the peripheral blood of asthmatic adults^6^, suggesting the potential roles of sialyl glycans in asthma. The relationships between the expression of adhesion-associated biomolecules on different T cells subsets with distinct trafficking potentials and asthma clinical parameters is therefore intriguing, and merits investigation.

Memory CD4 T helper cells^7^ and regulatory T cells^8^ play critical roles in the pathogenesis of asthma. There are two subsets of circulating memory T cells, with distinct functions: the central memory T (T_CM_) cells, and the effector memory T (T_EM_) cells^9^. T_CM_ cells, which express CCR7 and L-selectin, migrate to secondary lymphoid organs, while T_EM_ cells_,_ which lack CCR7 and L-selectin, preferentially migrate to peripheral tissues^9^. CCR4 appears to direct lung trafficking of pathogenic memory Th2 cell in asthma^3^, and is also required for the lung migration of Treg cells to attenuate allergic airway inflammation^5^. In asthmatic patients, increased numbers of CCR4^+^FOXP3^+^CD25^+^ Treg cells have been found in bronchoalveolar lavage after allergen challenge^10^. Improved characterization of the roles of the memory Th cells and Treg cells in asthma, especially their CCR7^+^ and CCR4^+^ subsets, could improve our understanding of asthma immunology.

Selectin-mediated cell adhesion is essential in lymphocyte homing and the recruitment of leukocytes to inflammation sites^11^. Sialyl Lewis X (sLe^X^), the glycan determinant that binds to E- and P-selectins^12^, is constitutively expressed on monocytes and granulocytes, and is suppressed on most resting lymphocytes^13^. Upon activation, however, resting T lymphocytes are induced to strongly express sLe^X^ ^13^. A recent study has also revealed the importance of sLe^X^ for identifying the most suppressive Treg cells^14^. Another carbohydrate ligand for all the selectin family members, sialyl 6-sulfo Le^X^, is known to be expressed on skin-homing central memory Th cells, and involved in the pathogenesis of immune-mediated diseases like atopic dermatitis^15^. Sialyl 6-sulfo Le^X^, however, loses its binding activity via cyclization of the sialyl acid moiety, transforming into cyclic sialyl 6-sulfo Le^X^ ^16^. Since our previous study found a significant increase in the proportion of sialyl 6-sulfo Le^X^ –expressing CCR4^+^ memory Th cells of asthma patients^6^, in the present study we assessed the expression of three carbohydrate glycans, sialyl 6-sulfo Le^X^, cyclic sialyl 6-sulfo Le^X^ and sLe^X^, on subsets of memory Th cells and Treg cells from the peripheral blood of asthmatic children, and examined their associations with asthma severity and clinical parameters in order to better understand their potential roles in the pathogenesis of asthma.

## Methods

### Study population

The Taiwanese Consortium of Childhood Asthma Study (TCCAS) is an ongoing, consortium-based study consisting of several paediatric study groups in Taiwan. In this study, TCCAS recruited Han Chinese asthmatic children aged 5 to 18 years old from the outpatient clinic of National Taiwan University Children Hospital and another paediatric clinic in 2015 and 2016. Asthma was diagnosed by a paediatric allergist or immunologist according to Global Initiative for Asthma (GINA) guidelines. Exclusion criteria included cancer, rare hereditary diseases, major immunological diseases, and states of severe infection. During enrollment, asthmatic children were required to have a 48-hour washout period, free of acute exacerbations. For asthmatic children, diagnosis and personal history of other allergic diseases such as allergic rhinitis, allergic dermatitis, and urticaria were also made and assessed by the specialist, according to clinical guidelines. We also enrolled healthy Han Chinese children of the same age range, with no history of physician-diagnosed asthma, allergic diseases, or other major diseases. All enrolled subjects and their parents provided their written informed consent during enrollment, and completed a detailed questionnaire. The questionnaire included items such as asthma daily symptoms, asthma onset time, family history, environmental data and the asthma control test (children’s version). Clinical parameters of the subjects, including asthma severity, pulmonary function and fractional exhaled nitric oxide (FeNO), were performed and acquired during enrollment. During the clinic visits, blood samples were also obtained and sent for analyses, which included biochemical testing, blood cell count, and cell staining. The study protocol was approved by the National Taiwan University Hospital Research Ethics Committee with the registration number #201302019RIND, which is in compliance with the Helsinki Declaration (Declaration of Helsinki, 2000) and the Belmont Report.

### Cell preparation and staining

Total blood cells (100 μl) were incubated for 30 min at 4 °C with primary antibodies. After two washes with PBS(-), the cells were incubated with FITC-conjugated secondary antibodies for 30 minutes at 4 °C. After hemolysis with FACS lysing solution (BD Biosciences) and two washes with PBS(-), the cells were subjected to flow cytometry analysis as described previously^17^.

The antibodies used included allophycocyanin (APC)-cyanin (Cy)7- conjugated anti-CD4 (RPA-T4), peridinin chlorophyll protein (PerCP)-Cy5.5-conjugated anti-CD25 (M-A251), phycoerythrin(PE)-Cy7-conjugated anti-CD45RO (UCHL1), Brilliant Violet 421-conjugated anti-CCR4 (L291H4), PE-conjugated anti-CCR7 (G043H7), FITC-conjugated anti-mouse IgG1 (PMG1-1), FITC-conjugated anti-mouse IgM (RMM-1) (BioLegend, San Diego, CA, USA), anti-human CD15s (CSLEX1(RUO)) (BD Pharmingen, San Jose, CA, USA), anti-sialyl 6-sulfo Le^x^ (G152, mouse IgM)^17^, and anti-cyclic sialyl 6-sulfo Le^x^ (G159, mouse IgG1)^17^. Leukocytes were treated with the FOXP3 Fix/Perm buffer set (BioLegend) to increase the permeability of cells, before being stained with Alexa Fluor 647-conjugated anti-FOXP3 (259D) (BioLegend).

### Flow cytometry analysis

**Figure 1:**
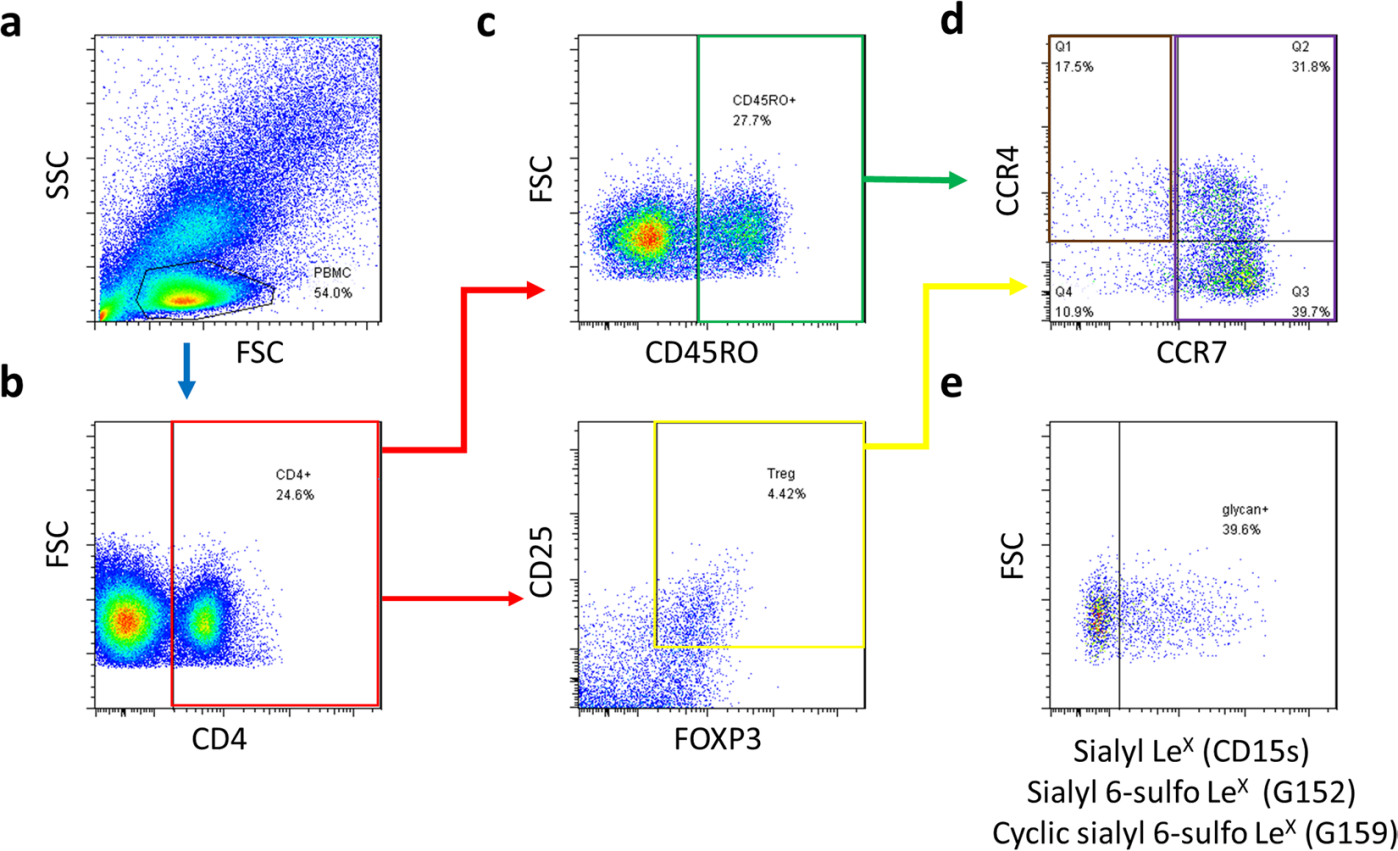
Gating strategy performed by flow cytometry. (**a**) PBMC were identified based on side scatter (SSC) and forward scatter (FSC). (**b**) CD4^+^ T cells were identified from PBMC based on CD4 expression. (**c**) Gating strategy for CD45RO^+^ memory Th cells (upper panel) and FOXP3^+^CD25^+^CD4^+^ Treg cells (lower panel), respectively. (**d**) CD4^+^ CD45RO^+^ memory Th cells and FOXP3^+^CD25^+^CD4^+^ Treg cells, respectively, were gated as CCR4^+^CCR7^−^ and CCR7^+^ subset. (**e**) Gating strategy for the cells which express sialyl Le^X^, sialyl 6-sulfo Le^X^, or cyclic sialyl 6-sulfo Le^X^.

Seven-colour immunofluorescence analysis was performed using a BD LSR II Flow Cytometer (BD Biosciences). Cell Quest software was used for collecting the fluorescent signal. The positive and negative populations were determined according to the staining of unreactive isotype-matched control IgG or IgM antibodies. The software program Flowjo (www.flowjo.com) was used for further analysis of cell subsets. The gating strategy for T cell subsets is shown in Fig. 1. Peripheral mononuclear cells (PBMC) were gated by forward scatter (FSC) and side scatter (SSC). CD4^+^ helper T (Th) cells were identified from PBMC based on CD4 expression. CD45RO^+^CD4^+^ memory Th cells and FOXP3^+^CD25^+^CD4^+^ regulatory T (Treg) cells, respectively, were gated based on respective cell markers from CD4^+^ Th cells. Both CD45RO^+^CD4^+^ memory Th and FOXP3^+^CD25^+^CD4^+^ Treg cells were classified into two subsets: the CCR4^+^CCR7^−^ subset, and the CCR7^+^ subset, according to the expression of two chemokine receptors, CCR4^9,18^ and CCR7^9^, with distinct homing potentials and effector functions. The percentages of cells which expressed sialyl glycans were calculated.

### Evaluation of asthma severity and clinical parameters

Asthma disease severity was determined according to 2015 GINA guidelines^19^, which divide patients into four severity grades: intermittent, mild, moderate, and severe based on daily symptoms, pulmonary function test and peak expiratory flow. Important asthma biomarkers such as peripheral blood eosinophil count, serum total IgE, pulmonary function test and FeNO were collected and used for further analysis.

### Statistical analysis

Demographic data was shown as means or median ± SD (standard deviation) or frequency. The Mann-Whitney U-test was used to assess the difference in the percentages of cells which express sialyl glycans between two different groups of subjects; the Kruskal-Wallis test and the post hoc Dunn’s test were performed by using the KW_MC SAS code^20^ for multiple group comparisons. Pearson and Spearman correlation coefficients were calculated to determine the associations between percentages of cells which express sialyl glycans and asthma biomarkers such as peripheral blood eosinophil count, the logarithm of serum total IgE, pulmonary function test and FeNO. SAS 9.3 software (SAS Institute, Cary, NC, USA) was used for statistical analysis. GraphPad Prism 7.0 (GraphPad Software, San Diego, CA, USA) was used to generate figures. P-values less than 0.05 were considered different for statistical significance.

### Exploratory gene expression analysis

We used publicly available Gene Expression Omnibus dataset GSE31773^21^ to explore gene expression of the enzymes related to the synthetic pathways of the three sialyl glycans-sialyl 6-sulfo Le^X^, cyclic sialyl 6-sulfo Le^X^, and sialyl Le^X^- from the CD4^+^ T cells of asthmatic adults and healthy controls. The bioinformatic analysis was performed following our previous study^22^ and the details could be found in Supplementary Table S1.

## Results

### Subject characteristics

We recruited 31 asthmatic children and 19 healthy controls in this study. The clinical characteristics of asthmatic and healthy subjects are shown in Table 1. Both the disease group and the reference control group have more than 10 subjects in each group, which is similar to the study design of previous studies of glycan expression in allergic diseases^6,23^. We enrolled asthmatic children with a wide spectrum of disease severity, ranging from intermittent to severe. For all asthmatic children, the average age of disease onset was 4.7 ± 1.7 years old; the average level of serum total IgE was 692.8 ± 832.9 IU/mL; the mean peripheral blood eosinophil count was 423 ± 274 cells/μL. Most asthmatic children had at least one other allergic disease.

**Table 1:** Clinical characteristics of asthma patients and healthy controls.

Variables	Healthy controls	Asthma patients
Number	19	31
Gender (male/female)	15/4	20/11
Age (mean ± SD)	11.5 ± 1.7	8.3 ± 3.1
Onset time (mean ± SD)	—	4.7 ± 1.7
Severity of asthma		
Intermittent	—	12
Mild	—	8
Moderate	—	8
Severe	—	3
Total IgE (mean ± SD) (IU/ml)	—	692.8 ± 832.9
Eosinophil blood count (mean ± SD) (cells/μL)	—	423 ± 274
Other allergic diseases		
Allergic conjunctivitis (yes/no)	—	12/19
Allergic rhinitis (yes/no)	—	24/7
Atopic dermatitis (yes/no)	—	6/25
Urticaria (yes/no)	—	5/26

### Significantly higher proportions of sialyl Le^X^ positive and sialyl 6-sulfo Le^X^ positive cells among memory T cells are detected in asthmatic children

**Figure 2:**
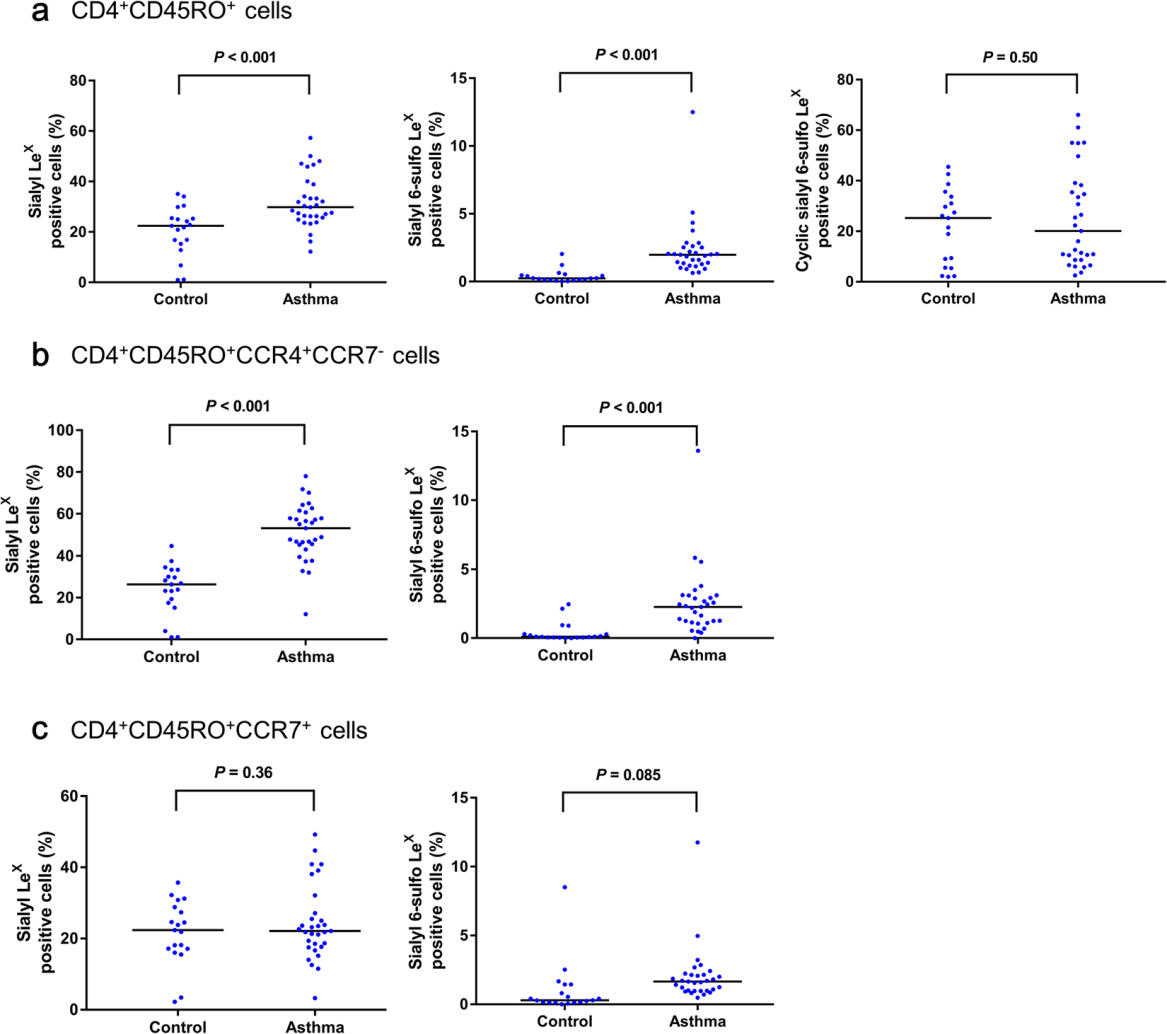
Comparison of sialyl Le^X^, sialyl 6-sulfo Le^X^, and cyclic sialyl 6-sulfo Le^X^ expression between healthy controls and asthmatic children. (**a**) The percentages of the cells which express sialyl Le^X^, sialyl 6-sulfo Le^X^, and cyclic sialyl 6-sulfo Le^X^ among CD4^+^CD45RO^+^ T cells. (**b**) The percentages of the cells which express sialyl Le^X^ or sialyl 6-sulfo Le^X^ among CCR4^+^CCR7^−^ memory Th cells. (**c**) The percentages of the cells which express sialyl Le^X^ or sialyl 6-sulfo Le^X^ among CCR7^+^ memory Th cells. Bars: median values.

We compared the expression of several sialyl glycans on memory helper T (Fig. 2) and suppressor T cells between patients and controls, to investigate their potential roles in asthma pathogenesis. We used G152, G159 and CD15s monoclonal antibodies to detect three sialyl glycans, – sialyl 6-sulfo Le^X^, cyclic sialyl 6-sulfo Le^X^ and sialyl Le^X^ – respectively. Our previous preliminary study revealed a significant increase in the proportion of sialyl 6-sulfo Le^X^ positive cells among CCR4^+^ memory Th cells from peripheral blood in patients with bronchial asthma^6^. We therefore compared the expression of sialyl Le^X^, sialyl 6-sulfo Le^X^, and cyclic sialyl 6-sulfo Le^X^ on all CD4^+^CD45RO^+^ memory Th cells, along with the CCR4^+^CCR7^−^ and CCR7^+^ subsets of memory Th cells, between asthmatic children (n = 31) and healthy controls (n = 19) (Fig. 2). On total memory Th cells, we detected significantly higher proportions of cells expressing sialyl Le^X^ and sialyl 6-sulfo Le^X^ in asthmatic children than healthy controls (p < 0.001 for both), with similar proportions of cyclic sialyl 6-sulfo Le^X^ positive cells between the two groups (p = 0.50).

Expression of sialyl 6-sulfo Le^X^ has been found on a specific subset of memory helper T cells expressing CCR4^24^. CCR4 is predominantly expressed on T helper 2 cells^25^, which are critical in asthma pathogenesis, and also important for T cell trafficking into the lung^18^. We investigated the sialyl glycan expression on two different subsets of CD4^+^CD45RO^+^ memory Th cells: effector memory Th cells, identified as CD4^+^CD45RO^+^CCR4^+^CCR7^−^, and lymph node-homing central memory Th cells, identified as CD4^+^CD45RO^+^CCR7^+^ ^5^. On the CCR4^+^CCR7^−^ subset of memory Th cells, we found higher ratios of cells which expressed both sialyl Le^X^ and sialyl 6-sulfo Le^X^ in asthmatic children than in healthy controls (p < 0.001 for both) (Fig. 2b); in contrast, on the CCR7^+^ subset of memory Th cells, we detected comparable percentages of both sialyl Le^X^- and sialyl 6-sulfo Le^X^-expressing cells between asthmatic children and healthy controls (p = 0.36 and 0.085, respectively) (Fig. 2c). These results suggest the potential roles of sialyl Le^X^ and sialyl 6-sulfo Le^X^ on the CCR4^+^CCR7^−^ subset of memory Th cells in the pathogenesis of asthma.

### Association between sialyl 6-sulfo Le^X^ on memory Th cells and peripheral eosinophil counts

**Figure 3:**
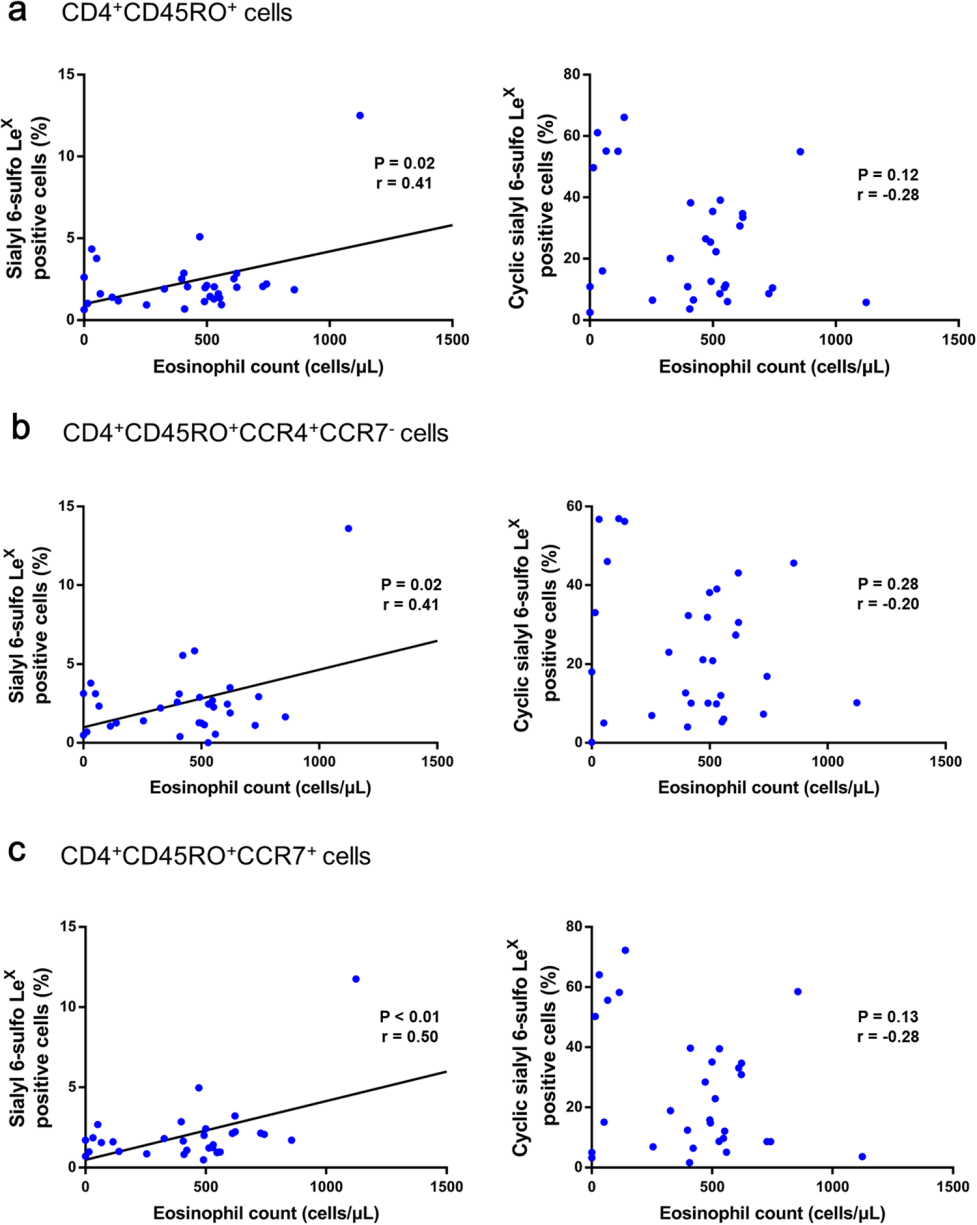
Association of sialyl Le^X^ and sialyl 6-sulfo Le^X^ expression on T cell subsets from asthmatic children with peripheral blood eosinophil count. The proportions of cells which express sialyl Le^X^ and sialyl 6-sulfo Le^X^ among CD4^+^CD45RO^+^ T cells (**a**), CCR4^+^CCR7^−^ (**b**), and CCR7^+^ (**c**) memory Th cells were analyzed using flow cytometry. Pearson correlation coefficients of the sialyl Le^X^ positive and the sialyl 6-sulfo Le^X^ positive CD4^+^CD45RO^+^ T cells (**a**), CCR4^+^CCR7^−^ (**b**), and CCR7^+^ (**c**) memory Th cells with peripheral blood eosinophil count were determined.

To explore the potential relationships between sialyl glycan expression and asthma biomarkers, we assessed the association between the proportions of cells which express sialyl glycans among memory Th cells and peripheral blood eosinophil counts, a biomarker strongly associated with exacerbations^26^ and treatment responses^27,28^. Surprisingly, peripheral blood eosinophil count was positively correlated with the expression of sialyl 6-sulfo Le^X^ on CD4^+^CD45RO^+^ memory Th cells, and the CCR4^+^CCR7^−^ and CCR7^+^ subsets of memory Th cells in asthmatic children (Fig. 3). In contrast, there was no significant association between peripheral blood eosinophil count and cyclic sialyl 6-sulfo Le^X^ on CD4^+^CD45RO^+^ memory Th cells, and the CCR4^+^CCR7^−^ and CCR7^+^ subsets of memory Th cells.

### Association between cyclic sialyl 6-sulfo Le^X^ on memory Th cells and serum total IgE

**Figure 4:**
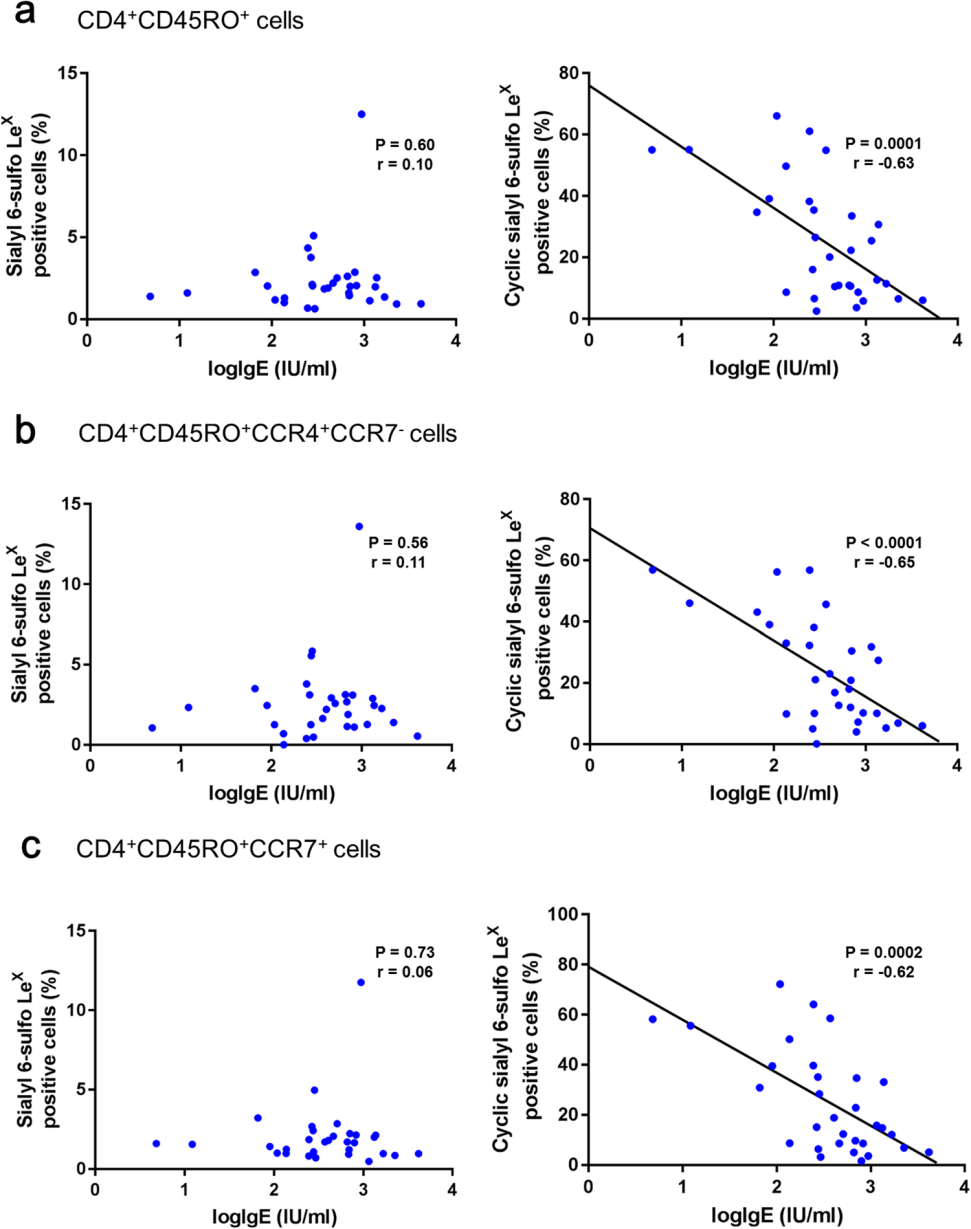
Association of sialyl Le^X^ and sialyl 6-sulfo Le^X^ expression on T cell subsets from asthmatic children with the logarithm of total IgE level (logIgE). The proportions of cells which express sialyl Le^X^ and sialyl 6-sulfo Le^X^ among CD4^+^CD45RO^+^ T cells (**a**), CCR4^+^CCR7^−^ (**b**), and CCR7^+^ (**c**) memory Th cells were analyzed using flow cytometry. Pearson correlation coefficients of the sialyl Le^X^ positive and the sialyl 6-sulfo Le^X^ positive CD4^+^CD45RO^+^ T cells (**a**), CCR4^+^CCR7^−^ (**b**), and CCR7^+^ (**c**) memory Th cells with the logarithm of serum total IgE were determined.

We further examined the association between serum total IgE, a biomarker for allergic immune responses, and the proportion of cells that express sialyl glycans among memory Th cells in asthmatic children. The logarithm of serum total IgE was negatively correlated with cyclic sialyl 6-sulfo Le^X^ positive cells among CD4^+^CD45RO^+^ memory Th cells, and the CCR4^+^CCR7^−^, and CCR7^+^ subsets of memory Th cells in asthmatic children (Fig. 4). In contrast, there was no significant association between the logarithm of serum total IgE and sialyl 6-sulfo Le^X^ positive cells among CD4^+^CD45RO^+^ memory Th cells, and the CCR4^+^CCR7^−^ and CCR7^+^ subsets of memory Th cells.

### A lower proportion of sialyl Le^X^ positive Treg cells is detected in asthmatic children

**Figure 5:**
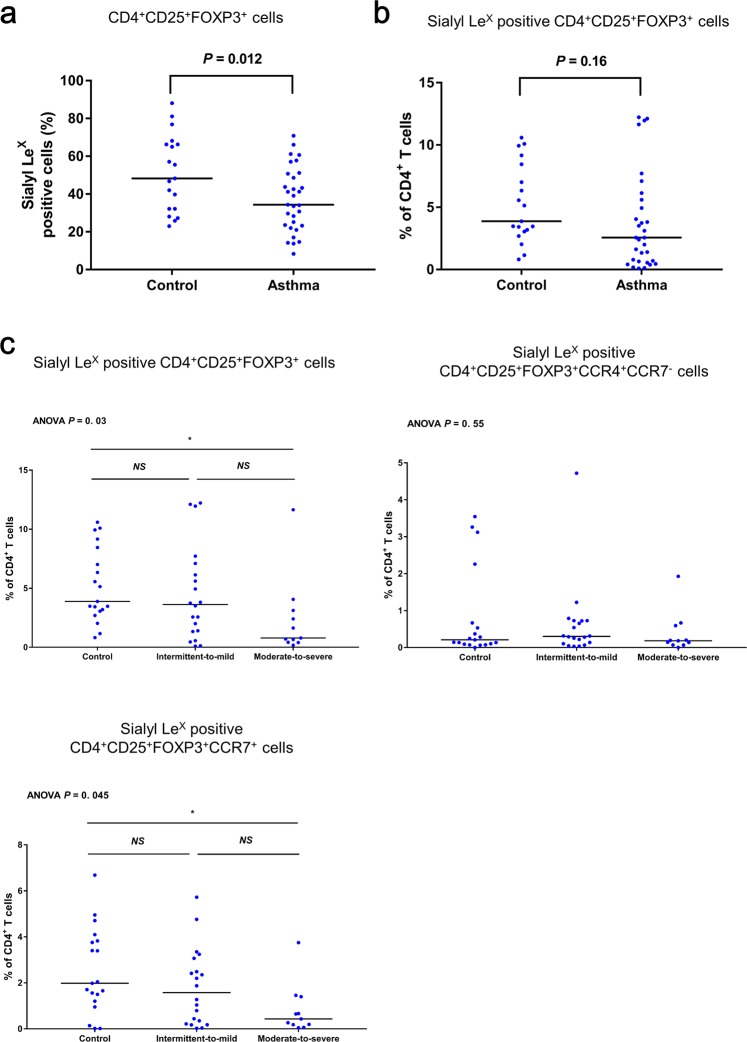
Association between sialyl Le^X^ positive Treg cells, and asthma status along with severity. (**a**) The proportions of sialyl Le^X^ positive cells among CD4^+^CD25^+^FOXP3^+^ Treg cells of asthmatic children and healthy controls. (**b**) The percentages of sialyl Le^X^ positive CD4^+^CD25^+^FOXP3^+^ Treg cells among CD4^+^ T cells of asthmatic children and healthy controls. (**c**) Association between the percentages of CD4^+^CD25^+^FOXP3^+^, CD4^+^CD25^+^FOXP3^+^CCR4^+^CCR7^−^, and CD4^+^CD25^+^FOXP3^+^CCR7^+^ Treg cells which express sialyl Le^X^ among CD4^+^ T cells with the severity of asthma. Non-parametric Kruskal-Wallis ANOVA was used to assess the differences among intermittent-to-mild (n = 20), and moderate-to-severe (n = 11) asthma and healthy controls (n = 19); post hoc Dunn’s test was utilized to test the differences between every two groups. Bars: median values; *post hoc p-value < 0.05; NS: post hoc p-value > 0.05.

FOXP3^+^ CD25^+^ CD4^+^ Treg cells (FOXP3^+^ Treg cells) have a key role in the regulation of allergen-triggered lung inflammation^29^; furthermore, sialyl Le^X^ is specifically expressed on the most suppressive FOXP3^+^ Treg cells in humans^14^. We compared the percentages of sialyl Le^X^ positive cells among FOXP3^+^ Treg cells between asthmatic children and healthy controls, and found that it was significantly lower in the asthma patients (p = 0.012) (Fig. 5a). We also compared the proportion of sialyl Le^X^ positive FOXP3^+^ Treg cells among total CD4^+^ T cells, and found that it was slightly lower in asthma children compared with healthy controls (p = 0.16), despite not reaching statistical significance (Fig. 5b).

### Association between sialyl Le^X^ positive Treg cells and the severity of asthma

To assess the association between CD15^+^ Treg cells and asthma, we compared the proportion of sialyl Le^X^ positive FOXP3^+^ Treg cells among CD4^+^ T cells between intermittent-to-mild (n = 20) and moderate-to-severe (n = 11) asthma and healthy controls (n = 19) (Fig. 5c). Statistical analysis showed decreased proportions of sialyl Le^X^ positive Treg cells in children with moderate-to-severe asthma compared with the healthy controls (post hoc p < 0.05). CCR4 is important for the migration of Treg cells to the lungs to attenuate allergic airway inflammation^4^, and CCR7 expression is required for Treg-mediated suppressive immune response in the priming phase^30^. We also compared the percentages of CCR4^+^CCR7^−^ and CCR7^+^ FOXP3^+^ Treg cells expressing sialyl Le^X^ among CD4^+^ T cells between patients with different severity and controls. No significant trend was found when we compared the percentages of sialyl Le^X^ positive FOXP3^+^CCR4^+^CCR7^−^ Treg cells among CD4^+^ T cells between two groups of asthmatic patients and healthy controls (Fig. 5c). On the contrary, the proportion of sialyl Le^X^ positive FOXP3^+^CCR7^+^CCR4^−^ Treg cells among CD4^+^ T cells was lower in moderate-to-severe asthma compared with healthy controls (post hoc p < 0.05) (Fig. 5c). These results suggest that children with moderate-to-severe asthma have lower proportions of suppressive sialyl Le^X^ positive FOXP3^+^ Treg cells, especially the CCR7^+^ subset with lymph node-homing ability, among peripheral CD4^+^ T cells.

### Exploratory gene expression analysis

The results of the gene expression comparison of five probes of three genes which are related to the synthetic pathways of sialyl 6-sulfo Le^X^, cyclic sialyl 6-sulfo Le^X^, and sialyl Le^X^ between the CD4^+^ T cells of severe asthmatic adults (n = 8) and those of healthy adults (n = 8) and their raw p-values without adjustment were shown in Supplementary Table S1.

## Discussion

In our study, we assessed the expression of sialyl carbohydrates on memory Th cells and Treg cells in childhood asthma. Results revealed higher proportions of cells expressing the sialyl Le^X^ and sialyl 6-sulfo Le^X^ among memory Th cells, especially on the CCR4^+^CCR7^−^ T_EM_ subsets in asthmatic children, compared with healthy controls. The sialyl 6-sulfo Le^X^ positive and the cyclic sialyl 6-sulfo Le^X^ positive memory Th cells were associated with absolute eosinophil count and IgE level in blood, respectively, which are important biomarkers in asthma. We also found a lower proportion of the sialyl Le^X^ positive cells among FOXP3^+^CD25^+^ Treg cells in asthmatic children, and significantly fewer numbers of sialyl Le^X^-positive Treg cells in children with moderate-to-severe asthma, in comparison with non-asthmatic children. These findings suggest the involvement of sialyl Le^X^, sialyl 6-sulfo Le^X^, and cyclic sialyl 6-sulfo Le^X^ in the pathogenesis of asthma. Associations between glycan expression and asthma clinical parameters, such as disease severity and blood biomarkers, may also reflect the influences of these biomolecules on asthma.

Our study implicated two sialyl glycans, sialyl Le^X^, and sialyl 6-sulfo Le^X^, in the mediation of the function of memory Th cells in asthma. We found higher ratios of cells expressing these two glycans among memory Th cells in the peripheral blood of asthmatic children, compared with healthy controls (Fig. 2a). Previous studies have shown that T helper 1 (Th1) cells preferentially express sialyl Le^X^ upon activation, compared with Th2 cells^31^. Therefore, higher proportions of activated sialyl Le^X^ positive memory Th cells may indicate the possible activation of Th1 immune responses in our asthmatic children. We also found that the differences of sialyl Le^X^ positive and sialyl 6-sulfo Le^X^ positive cells among memory Th cells in childhood asthma exist predominantly in CCR4^+^CCR7^−^ effector memory Th subset, instead of in CCR7^+^ central memory Th subset (Fig. 2b,c). In asthma, CCR4 expression is associated with lung trafficking potential of pathogenic memory Th2 cell and can be found on most lung-homing tissue-resident memory T cells^3,32^. Besides, sialyl 6-sulfo Le^X^ was found to be preferentially expressed on the endothelium of lung tissues from asthma patients^33^. Further studies are required to understand whether sialyl 6-sulfo Le^X^ expression is involved in determining the lung homing ability of effector memory Th2 cells and tissue-resident memory T cells in asthma.

We found that sialyl 6-sulfo Le^X^ positive cells among memory Th cells were positively correlated with the absolute eosinophil count of peripheral blood in asthmatic children (Fig. 3). The peripheral blood eosinophil count is a marker of eosinophilic inflammation, and is highly associated with exacerbation^26^ and treatment response^27,28^. The proportion of cyclic sialyl 6-sulfo Le^X^ positive cells among memory Th cells was negatively correlated with the logarithm of serum total IgE level, another important biomarker^34^, in asthmatic children (Fig. 4). Both serum total IgE and eosinophil count are important clinical parameters of asthma. Cyclic sialyl 6-sulfo Le^X^ is synthesised from sialyl 6-sulfo Le^X^ by sialic-acid-cyclizing-enzymes, such as sialic acid de-N-acetylase and cyclase; cyclic sialyl 6-sulfo LeX could also be reconverted back to sialyl 6-sulfo Le^X^ by acid-decyclizing-enzymes, such as sialic acid hydrolase and N-acetyltransferase^17^. Altered activities of sialyl acid cyclizing and decyclizing enzymes in memory helper T cells might be a possible explanation behind our observation, and would be explored in our future study. Our findings suggest that sialyl 6-sulfo Le^X^, and cyclic sialyl 6-sulfo Le^X^ expression might have a role in determining the function of memory Th cells in Th2 inflammation, thus affecting clinical presentations of asthma.

Our study revealed the importance of sialyl Le^X^ positive FOXP3^+^CD25^+^ Treg cells in asthma. We found that asthmatic children had a lower proportion of the sialyl Le^X^ positive cells^14^ among total FOXP3^+^CD25^+^ Treg cells from peripheral blood (Fig. 5a). Although the numbers of sialyl Le^X^ positive FOX3^+^ CD25^+^ Treg cells among CD4^+^ cells did not reach statistical significance between children with mild-to-intermittent asthma and healthy controls, children with moderate-to-severe asthma had a lower proportion of both the total cells and the CCR7^+^ subset of sialyl Le^X^ positive FOX3^+^ CD25^+^ Treg cells, among CD4+ T cells (Fig. 5c). Treg cells play key roles in promoting and sustaining tolerance to allergens by regulating allergen-triggered immune responses, and a failure to develop that tolerance could lead to the emergence of a pathologic Th2 response^29^. A decreased proportion of sialyl Le^X^ positive Treg cells in the blood of moderate-to-severe asthmatic children may indicate attenuated suppression of Th2 immune response mediated by Treg cells in those patients. CCR7 is required for Treg cells to suppress allergic airway inflammation during the sensitization phase^10^. Isolation of total and the CCR7^+^ subset of sialyl Le^X^ positive FOX3^+^ CD25^+^ Treg cells in the airway specimens of asthma patients and utilization of *in vivo* model would help further clarify the potential role of sialyl Le^X^ on Treg cells in asthma.

We also sought to assess the associations between sialyl 6-sulfo Le^X^ and cyclic sialyl 6-sulfo Le^X^ glycans on memory Th cells and other asthma biomarkers. Pulmonary indicators, such as forced vital capacity (FVC) and forced expiratory volume in one second (FEV1), are commonly used to evaluate the response of asthma treatment. However, both these indicators and fractional exhaled nitric oxide (FeNO) were poorly associated with the expression of sialyl glycans (data not shown). We also investigated the potential association between all above-mentioned biomarkers of asthma with sialyl glycans expression on FOXP3^+^ Treg cells, which showed poor association (data not shown). We have explored gene expression of the enzymes involved in the synthetic pathways of these sialyl glycans by using the publicly available Gene Expression Omnibus datasets, and found a trend of altered gene expression of these enzymes in the CD4^+^ T cells of patients with severe asthma (Supplementary Table S1), which might indicate the potential underlying mechanisms behind our observations. We would continue our investigations of the mechanistic alterations in our future study.

To the best of our knowledge, this is the first study to investigate the potential roles of the sialyl carbohydrates sLe^X^, sialyl 6-sulfo Le^X^, and cyclic sialyl 6-sulfo Le^X^ on memory Th cells and Treg cells in asthmatic children. However, it is limited in several ways. This is a cross-sectional study; we plan to follow up with these asthmatic children longitudinally to assess potential relationships between sialyl glycan expression and clinical outcomes, such as treatment responses and exacerbations. We did not perform *ex vivo* assays, to assess the functions and cytokine profiles of isolated T cell subsets after allergen stimulation. Examining sialyl glycan expression on central and memory Th2 cells could further clarify the effect of these molecules during interactions between immune cells and/or membranes in asthma. Also, our study specimens were peripheral blood. This is easier to acquire in clinical settings compared with procedures such as induced sputum and bronchoscopy, and identification of biomolecules in blood associated with current status of asthma may facilitate the development of convenient diagnostic tools. On the other hand, asthma is an airway inflammatory disease; and acquisition of airway samples could better inform the role of the sialyl carbohydrates for lung trafficking of T cells.

In this study, we found higher proportions of cells which express sialyl Le^X^ and sialyl 6-sulfo Le^X^ on memory Th cells in asthmatic children than non-asthmatic children, a trend that seems to depend on CCR4^+^CCR7^−^ T_EM_ cells with lung migration tendency. The results also show a decreased proportion of the sialyl Le^X^ positive Treg cells in children with moderate-to-severe asthma, and correlations between sialyl 6-sulfo Le^X^ and cyclic sialyl 6-sulfo Le^X^ expression with blood biomarkers. These findings also suggest the likelihood of involvement of sialyl 6-sulfo Le^X^ and cyclic sialyl 6-sulfo Le^X^ in Th2 inflammation, thus affecting different clinical manifestations. It is necessary to link sialyl glycan expression on T cell subsets to treatment responses and asthma subtypes in future studies, which may provide useful markers or even novel therapeutic targets in asthma treatment.

## Supplementary information

Supplementary Table S1
