# Supplementary material for: Sialyl Glycan Expression on T Cell Subsets in Asthma: a correlation with disease severity and blood parameters
Source: Sci Rep. 2019 Jun 20;9:8947. doi: 10.1038/s41598-019-45040-2 (PMC6586815; doi:10.1038/s41598-019-45040-2)
Supplement: Supplementary file 1 — Supplementary Table S1 [file 41598_2019_45040_MOESM1_ESM.pdf]

Sialyl Glycan Expression on T Cell Subsets in Asthma: a correlation with disease severity and blood parameters

Yu-Liang Yeh[1,2,3], Wen-Chia Wu[1], Reiji Kannagi[2], Bor-Luen Chiang[4], Fu-Tong Liu[2], Yungling Leo Lee[1,2*]

Supplementary Table S1. The trend of altered gene expression of several glycosyltransferases in CD4$^+$ T cells from adults with severe asthma (n=8), compared with healthy controls (n=8) was shown. We performed the exploratory analysis by using the microarrays from the publicly available Gene Expression Omnibus (GEO) dataset GSE31773[1]. To explore the gene expression of the enzymes that are involved in the synthetic pathways of the three sialyl glycans-sialyl 6-sulfo Le^X, cyclic sialyl 6-sulfo Le^X, and sialyl Le^X-in our study, we compared the expression levels of several genes from the CD4$^+$ T cells between adults with severe asthma (n=8), and healthy controls (n=8). The results were shown below, and the bioinformatic analysis was performed following the protocols of our previous study[2]. 6-O-sulfotransferase, beta 1,4- galactosyltransferase, alpha-2,3-sialyltransferase are three important enzymes that are involved in the pathways of these three sialyl glycans[3]. As shown in the table below, we found that there were some trends of altered gene expression of these three enzymes involved in the glycan synthesis in CD4$^+$ T cells in severe asthma, despite the small fold changes and the p values without adjusting for multiple testing problems.

Symbol	Name	Fold	P-value
HS6ST1	heparan sulfate 6-O-sulfotransferase 1	1.27	0.035
B4GALT2	UDP-Gal:betaGlcNAc beta 1,4- galactosyltransferase, polypeptide 2	1.30	0.032
B4GALT5	UDP-Gal:betaGlcNAc beta 1,4- galactosyltransferase, polypeptide 5	0.61	0.006
B4GALT6	UDP-Gal:betaGlcNAc beta 1,4- galactosyltransferase, polypeptide 6	1.59	0.024
ST3GAL3	ST3 beta-galactoside alpha-2,3-sialyltransferase 3	1.54	0.015

The raw p-values were shown without adjustment.

Fold: the log2 fold of gene expression from CD4$^+$ T cells of adults with severe asthma compared with healthy controls.

REFERENCE

1. Tsitsiou, E. *et al*. Transcriptome analysis shows activation of circulating CD8+ T cells in patients with severe asthma. *J. Allergy Clin. Immunol*. **129**, 95-103 (2012).
2. Yeh, Y. L. *et al*. Genetic profiles of transcriptomic clusters of childhood asthma determine specific severe subtype. *Clin. Exp. Allergy*. **48**, 1164-1172 (2018).
3. Sakuma, K. *et al*. Sialic acid cyclization of human Th homing receptor glycan associated with recurrent exacerbations of atopic dermatitis. *J. Dermatol. Sci.* **68**, 187-193 (2012).
